# Functional Materials from Biomass-Derived Terpyridines: State of the Art and Few Possible Perspectives

**DOI:** 10.3390/ijms25169126

**Published:** 2024-08-22

**Authors:** Jérôme Husson

**Affiliations:** Institut UTINAM, UMR CNRS 6213, Université de Franche-Comté, 16 Route de Gray, F-25000 Besançon, France; jerome.husson@univ-fcomte.fr

**Keywords:** catalysts, dye-sensitized solar cells, ligands, metal complexes, metal–organic frameworks, oligopyridines

## Abstract

This review focuses on functional materials that contain terpyridine (terpy) units, which can be synthesized from biomass-derived platform chemicals. The latter are obtained by the chemical conversion of raw biopolymers such as cellulose (e.g., 2-furaldehyde) or lignin (e.g., syringaldehyde). These biomass-derived platform chemicals serve as starting reagents for the preparation of many different terpyridine derivatives using various synthetic strategies (e.g., Kröhnke reaction, cross-coupling reactions). Chemical transformations of these terpyridines provide a broad range of different ligands with various functionalities to be used for the modification or construction of various materials. Either inorganic materials (such as oxides) or organic ones (such as polymers) can be combined with terpyridines to provide functional materials. Different strategies are presented for grafting terpy to materials, such as covalent grafting through a carboxylic acid or silanization. Furthermore, terpy can be used directly for the elaboration of functional materials via complexation with metals. The so-obtained functional materials find various applications, such as photovoltaic devices, heterogeneous catalysts, metal–organic frameworks (MOF), and metallopolymers. Finally, some possible developments are presented.

## 1. Introduction

**Figure 1:**
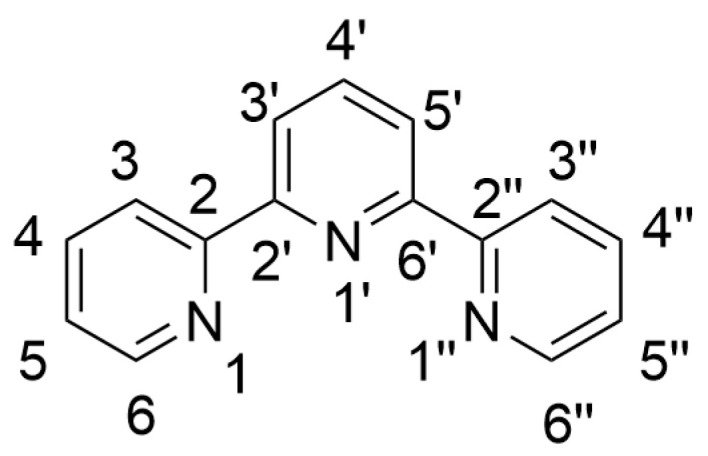
Chemical structure and atom-numbering of 2,2′:6′,2″-terpyridine.

In 1932, Morgan and Burstall [1] described the first synthesis of 2,2′:6′,2″-terpyridine (Figure 1). Since then, a plethora of terpyridine derivatives has been prepared. This can be explained by the fact that terpyridine derivatives (terpys) are ligands able to form complexes with various metals. Varying the nature of the metallic center and/or substituents onto the ligands offers the possibility to prepare a broad range of different substances. The so-obtained compounds find applications in numerous fields [2,3], such as in medicinal chemistry [4], biological probes [5], sensors [6], or as energy storage devices [7], just to name a few.

In the late 1990s, Anastas and Warner introduced the twelve principles of green chemistry as guidelines to make chemical processes more environmentally friendly [8]. One of the principles is to use renewable feedstocks, such as biomass, instead of oil-derived substances. In fact, biomass is renewable, contrary to crude oil. Furthermore, the use of crude oil has detrimental effects on the environment [9]. Consequently, turning to biomass as a source of chemicals can help mitigate environmental problems [10]. This review emphasizes using biomass-derived platform chemicals in synthesizing terpyridine ligands as a green chemistry approach. The utilization of these ligands in the fabrication of functional materials is also presented. Finally, applications of the so-obtained materials are described.

## 2. Preparation of Functional Materials from Biomass-Derived Terpyridines

**Scheme 1:**
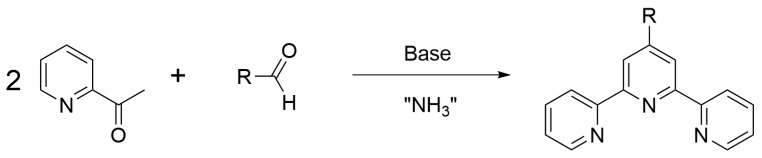
Reaction scheme for the Kröhnke synthesis of terpyridines.

Many synthetic pathways are available for the preparation of terpyridine derivatives [11,12,13]. Most of them rely on the Kröhnke reaction (Scheme 1), which involves the reaction of two equivalents of an acetylpyridine derivative with an aldehyde in a basic medium and in the presence of an ammonia source [14].

**Figure 2:**
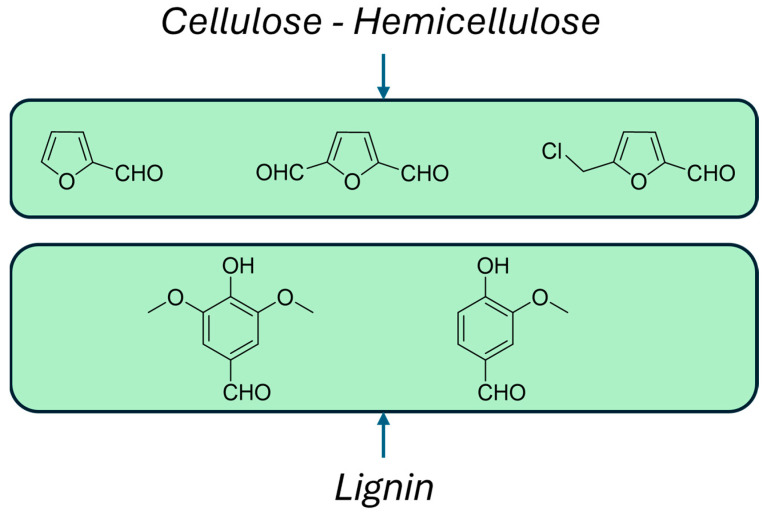
Selected examples of biomass-derived aldehydes obtained from cellulose, hemicellulose, or lignin.

Many aldehydes (Figure 2) are available through the conversion of biomass [15,16]. For instance, furan-derived aldehydes (2-furaldehyde, 2,5-diformylfuran, 5-halomethylfurfural, etc.) are obtained by chemical conversion of cellulose or hemicellulose [17,18,19,20,21,22,23,24], while vanillin and syringaldehyde derivatives can be obtained by the transformation of lignin [25,26,27,28,29,30,31].

Therefore, it is no surprise that biomass-derived aldehydes can be used as starting materials in the preparation of terpys to be used in functional materials.

### 2.1. Materials for Photovoltaic Applications

**Figure 3:**
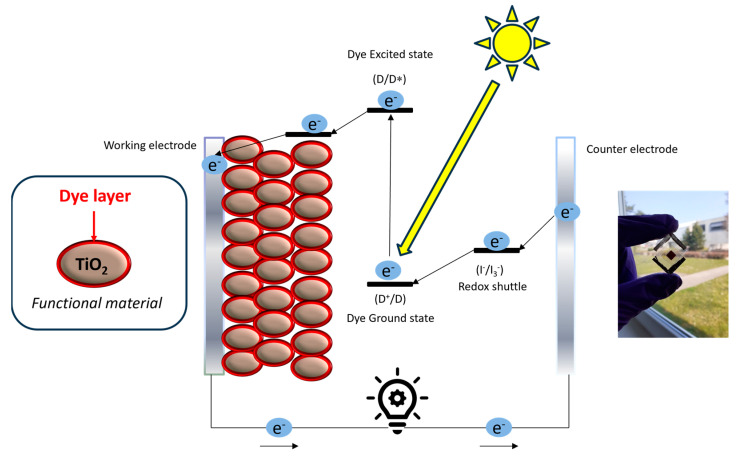
Schematic representation of a DSSC.

Dye-sensitized solar cells (DSSC) are devices that turn solar light into electricity [32]. Firstly, described in 1991 [33], these photoelectrochemical systems rely on a functional material [34], which combines a wide bandgap semiconductor (generally TiO_2_) and an organic or organometallic sensitizer (the “dye”). Classically, such solar cells are composed of two electrodes, one of them being coated with the functional material (Figure 3).

Upon light irradiation, the dye (D) undergoes an excited state (D*) from which an electron is injected onto the semiconductor, thus generating the electrical current and leaving the sensitizer in an oxidized state. This requires the sensitizer to be regenerated in its ground, reduced state by recovering an electron from the counter-electrode. To achieve this, a redox shuttle (I_3_^−^/I^−^, for example) is used to transfer electrons from one side of the system to another. This closes the cycle, which can be repeated [35]. The efficiency of such solar cells (η) is defined as the ration between the energy received from light and the electrical energy generated by the system. This efficiency is expressed as a percentage, and currently, the most efficient devices have η around 13% [36].

**Figure 4:**
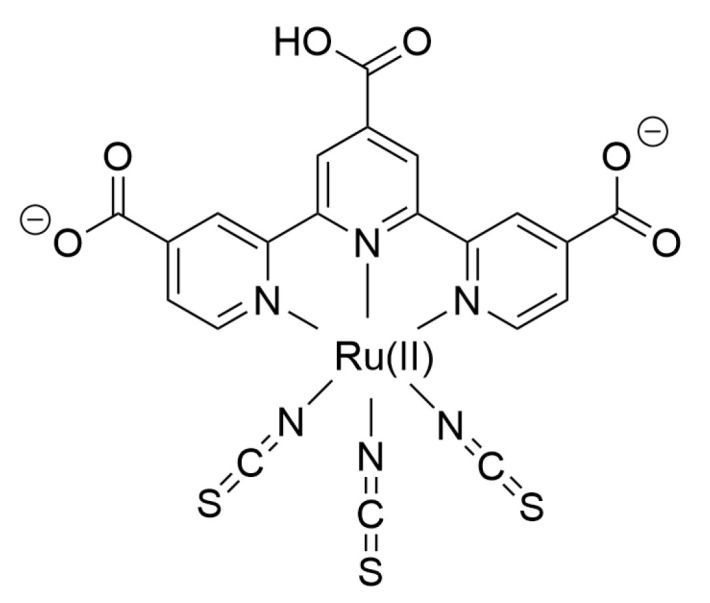
Chemical structure of “Black-Dye” (**1**). Tetrabutylammonium counter-cations omitted.

Amongst the variety of sensitizers that have been evaluated in DSSCs [37], terpyridines play an important role [38,39]. For instance, the so-called “black-dye” (**1**) (Figure 4) has been, for many years, one of the most efficient sensitizers [40,41].

**Scheme 2:**
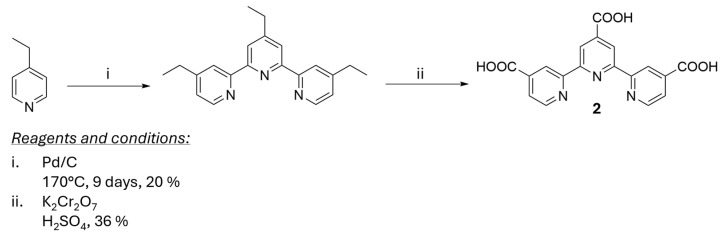
Original route to 4,4′,4″-tricarboxy-2,2′:6′,2″-terpyridine (**2**).

This ruthenium (II) terpyridine complex features carboxylate groups, which ensure a good anchorage onto TiO_2_ nanoparticules, as well as favoring electron injection, thus forming a valuable functional-material for DSSC applications [42]. Preparing “black-dye” requires the prior synthesis of 4,4′,4″-tricarboxy-2,2′:6′,2″-terpyridine ligand (**2**). The original route towards compound **2** is based on a two-step process (Scheme 2) from 4-ethylpyridine. Nevertheless, this pathway suffers low yield, high energy demand, usage of toxic reagents, and a high level of chemical waste.

**Scheme 3:**
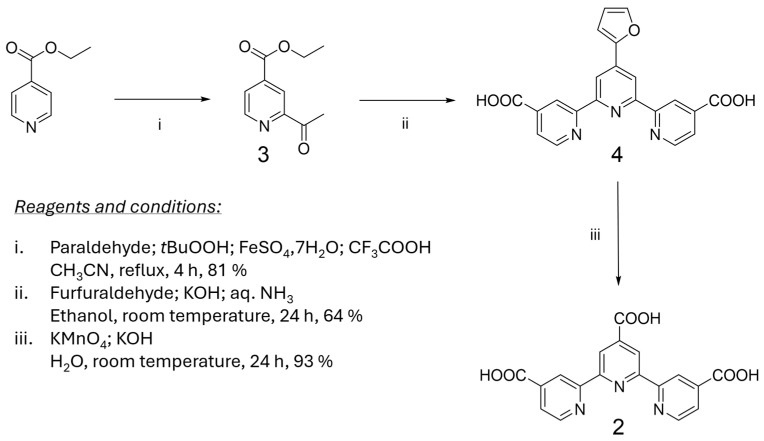
“Furan” alternative route to 4,4′,4″-tricarboxy-2,2′:6′,2″-terpyridine.

Alternatively, molecule **2** can be prepared from biomass-derived furfural in three steps, as depicted in Scheme 3 [43,44]. The initial step consists on the synthesis of keto-derivative (**3**) from commercially available ethyl isonicotinale and a Minisci-type reaction [45]. The former is then converted to furan-substituted terpy **4** through a reaction with furfural under Kröhnke’s conditions. Finally, the furan ring is degraded into carboxylate using a potassium permanganate-mediated oxidation.

When compared to the original route to (**2**), the “furan” alternative ticks several principles of green chemistry [46]:

- 1) Prevention of wastes;
- 3) Less hazardous chemical synthesis;
- 6) Design for energy efficiency;
- 7) Use of renewable feedstock.

In fact, the amount of waste generated during the process, as measured by the E-factor [47], is divided by a factor of approximately three (106 vs. 336 g.g^−1^, respectively). Secondly, the furan route avoids the use of toxic chromium (VI) salts. Thirdly, this process is less energy-intensive, and finally, it uses a biomass-derived reagent (furfural). With this new, more environmentally friendly method for the preparation of ligand **2**, many other metal complexes incorporating it were synthesized and evaluated in DSSCs [48,49,50,51,52].

**Scheme 4:**
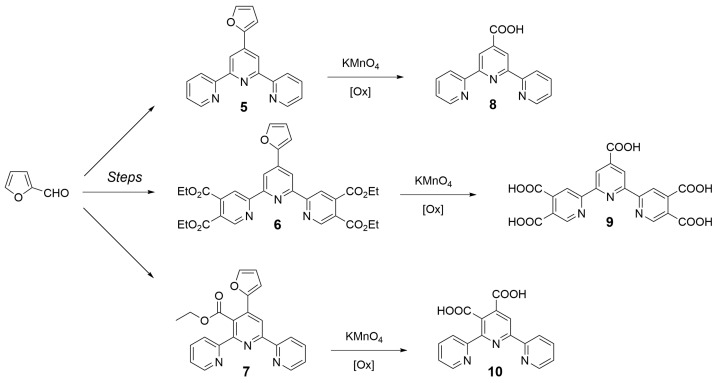
Synthetic pathways for the preparation of ligands **5**–**10** from biomass-derived furfural.

Biomass-derived furfural can also be used for the preparation of terpyridines **5**–**7**, which can undergo oxidation of the furan ring to carboxylic acid moieties similarly to the preparation of **2** [43,53,54]. This provides new ligands **8**, **9,** and **10** (Scheme 4), which can be incorporated into various metal-based sensitizers for DSSCs [55,56,57,58,59].

**Scheme 5:**
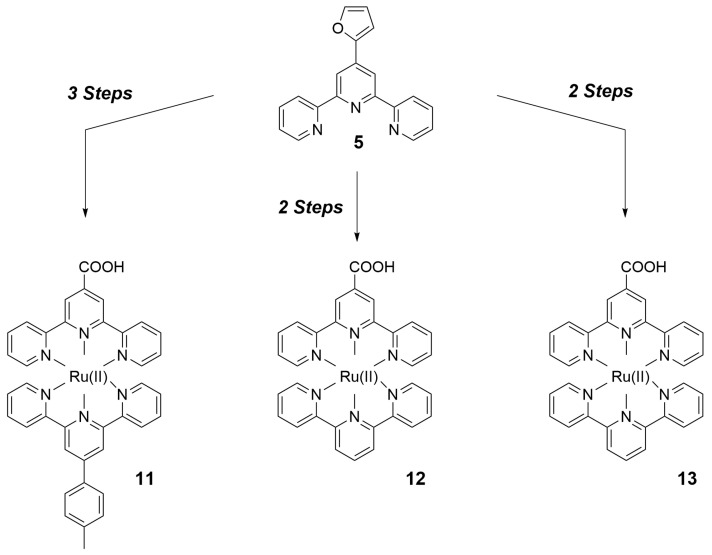
Conversion of biomass-derived terpyridine **5** into sensitizers **11**–**13** (counter-anions omitted).

It is worth mentioning that the furan-oxidation methodology can be extended to the preparation of ligands for the fabrication of sensitizers that do not contain terpyridines, but bipyridine or bipyrimidine instead [60,61,62]. Moreover, furan oxidation can be carried out directly onto a preformed complex [63], thus allowing the synthesis of dyes **11**–**13** (Scheme 5) starting from biomass-derived terpy **5** [64,65,66].

**Figure 5:**
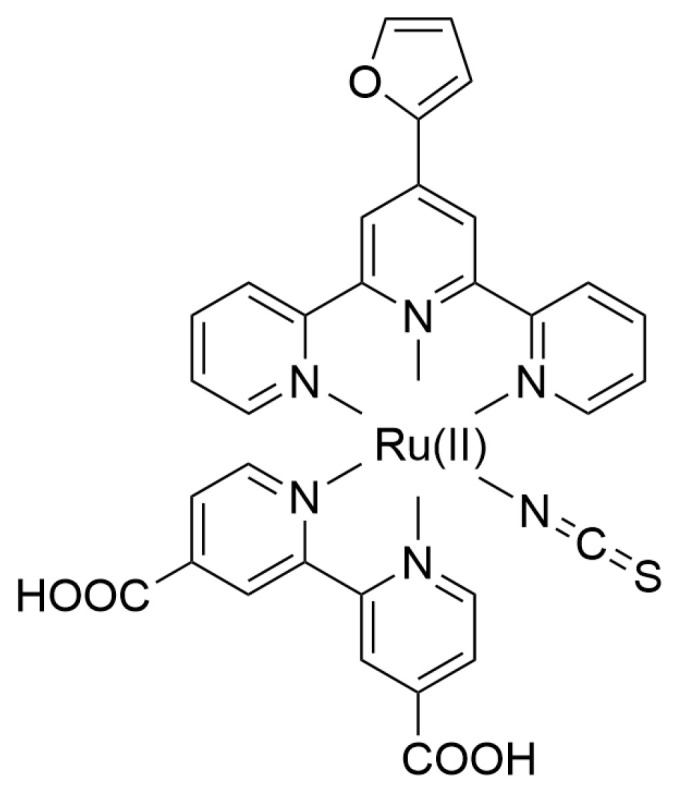
Chemical structure of complex **14**.

Apart from being an important intermediate in the preparation of carboxy-substituted dyes, terpyridine **5** was also used for the preparation of metal-based sensitizers in which the furan heterocycle is unaltered. Molecule **5** was combined with 4,4′-dicarboxy-2,2′-bipyridine to afford heteroleptic complex **14** (Figure 5), which was assessed as a dye in DSSCs. Anchorage onto TiO_2_ is effected via the bipyridine part of the complex thanks to -COOH groups. Unfortunately, the efficiency of the device so-fabricated is limited to a poor 0.13% [67].

**Figure 6:**
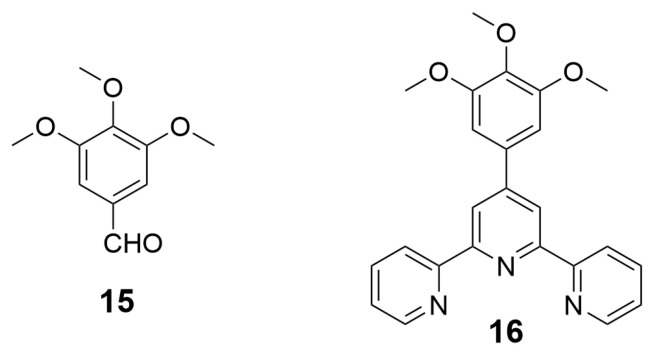
Chemical structures of 3,4,5-trimethoxybenzaldehyde (**15**) and 4′-(3,4,5-trimethoxypenyl)-2,2′:6′,2″-terpyridine (**16**).

As can be seen, furfural has been widely used in the preparation of terpyridine-based dyes. Nevertheless, other biomass-derived chemicals have also been used in the conception of such sensitizers. For instance, 3,4,5-trimethoxybenzaldehyde (**15**) can be used to prepare terpyridine **16** (Figure 6) using the Kröhnke reaction [68]. 3,4,5-trimethoxybenzaldehyde can be obtained from naturally occurring syringaldehyde, vanillin, or gallic acid [69,70,71]. Thus, one can consider ligand **15** as a biomass-derived compound.

**Scheme 6:**
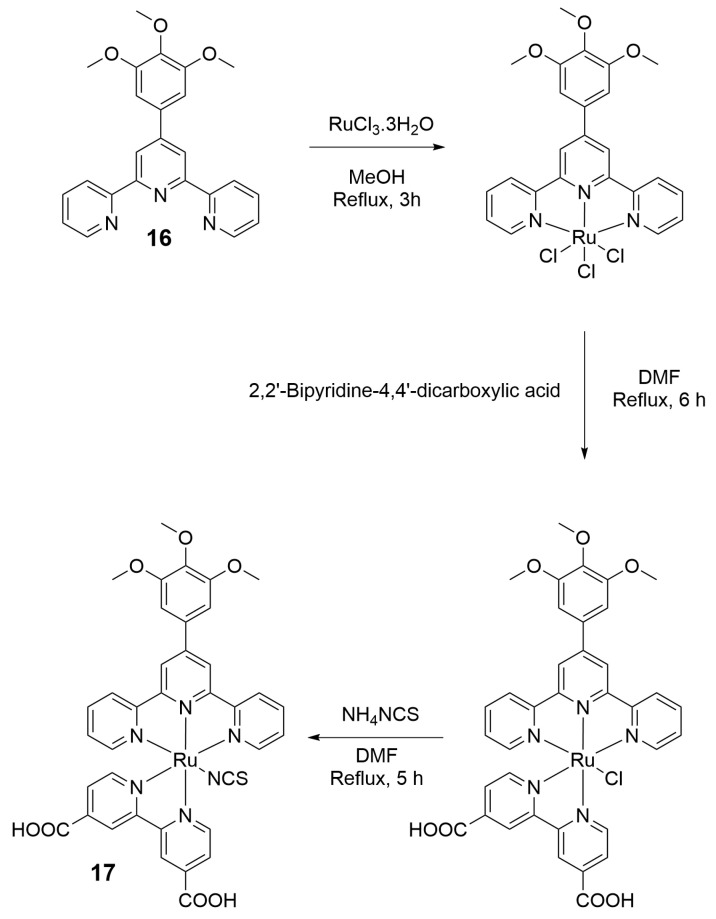
Synthetic pathway towards photosensitizer **17**.

Terpyridine **16** is then converted in three steps to sensitizer **17** (Scheme 6). This heteroleptic Ru(II) complexes is then adsorbed onto TiO_2_, and the obtained functional material is evaluated in a DSSC. The device reaches 4.62% efficiency [72].

**Scheme 7:**
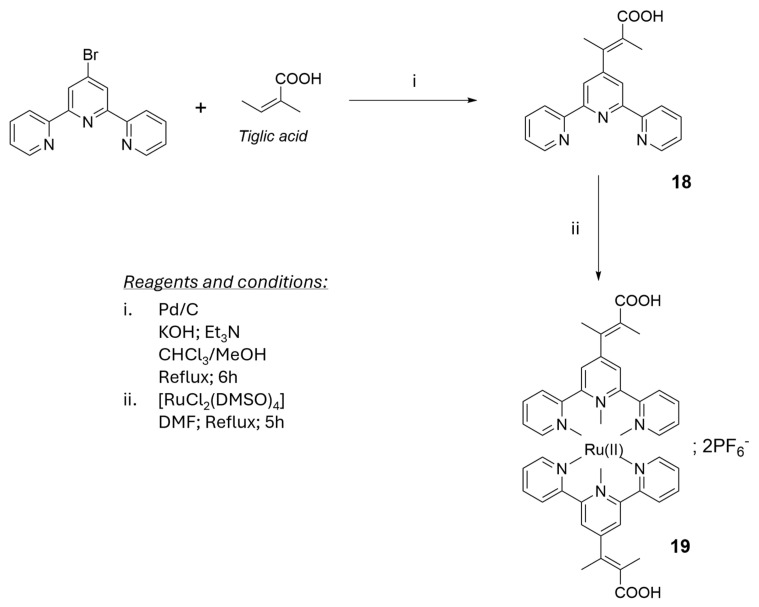
Synthetic pathway towards complex **19**.

In the above-mentioned examples, all sensitizers are prepared from aldehydes via the Kröhnke reaction. However, it is also possible to prepare dye from non-aldehyde biomass-derived platform chemicals, such as Tiglic acid, a naturally occurring substance that can be isolated from many different plants [73]. Heck cross-coupling reaction with 4′-bromo-2,2′:6′,2″-terpyridine afforded ligand **18**. The latter is used for the synthesis of Ru(II)-homoleptic complex **19** (Scheme 7). Although no solar cell was made and characterized in the study, the photophysical properties of **19** were studied and suggested a possible application in DSSCs owing to its high molar absorption coefficient [74].

### 2.2. Materials for Catalysis

Terpyridine derivatives are widely employed in the field of catalysis [3,75]. Functional materials that incorporate biomass-derived terpyridines have been investigated as catalysts, especially in heterogeneous catalysis. In fact, such functional materials are generally insoluble in the reaction medium, thus allowing their recovery at the end of the reaction.

**Figure 7:**
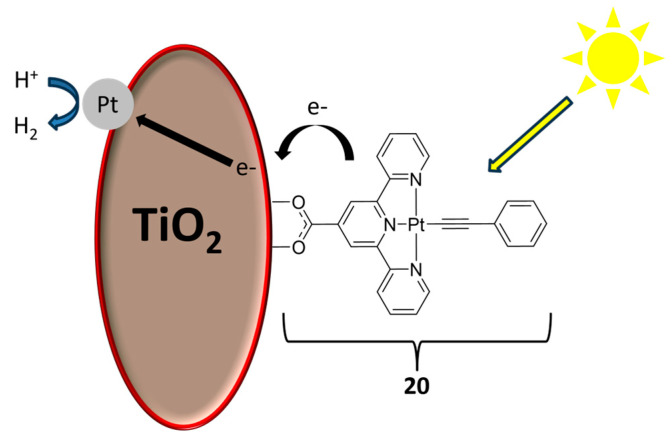
Schematic representation of the photocatalyst based on complex **20**.

Quite similar to DSSCs (see above Section 2.1), complex **20** (Figure 7) can be grafted onto platinized titanium dioxide. The obtained functional material can be used as a photocatalyst for the generation of H_2_ in aqueous media [76]. Upon irradiation, the Pt-complex injects an electron onto TiO_2_. This photogenerated electron is driven to Pt-particules, where it achieves proton reduction to H_2_. Regeneration of the sensitizer to its ground state is achieved via a sacrificial electron donor (triethanolamine).

This material is able to produce H_2_ upon irradiation at λ > 410 nm but with low rates and few turnovers. A possible explanation for these facts is the oxidative degradation of the complex onto the surface.

**Figure 8:**
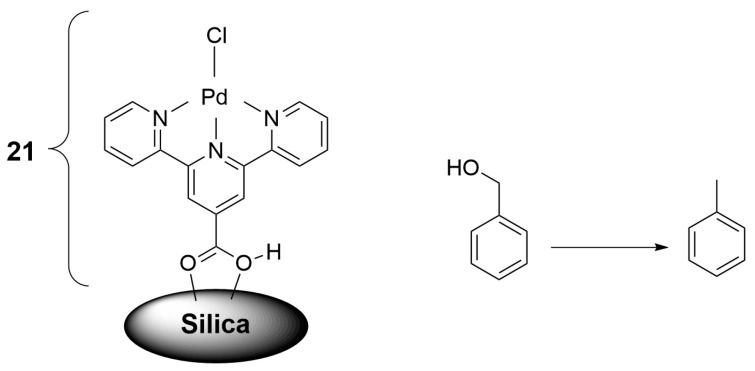
Structure of heterogenous catalyst (**left**) based on complex **21** and the model hydrodeoxygenation reaction (**right**) that it catalyzes.

Terpyridine **8** is also used to prepare complex **21** (Figure 8). Thanks to the carboxylic acid group, compound **21** can be anchored onto silica, thus making a heterogenous catalyst that can be used for the hydrodeoxygenation of aromatic alcohols [77]. Different binding modes are proposed between the molecular part of the catalyst and the silica material. These include electrostatic interactions, hydrogen, and covalent bondings.

This catalyst is easily prepared by simply soaking silica particles in a DMF solution of the complex. This point is advantageous due to potential large-scale applications. The efficiency of this catalyst in the model reaction was measured in different solvents ranging from polar ones (e.g., methanol) to apolar ones (e.g., dodecane). Best efficiencies are achieved in non-polar solvents. In fact, such solvents are able to stabilize catalyst bonding onto silica. Furthermore, these solvents do not coordinate with the Pd-center, thus preserving catalyst efficiency.

**Figure 9:**
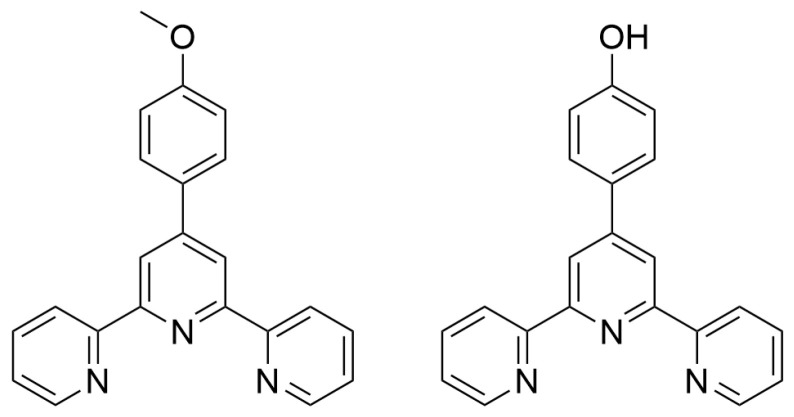
Chemical structures of terpyridine **22** (**left**) and **23** (**right**) synthesized from anisaldehyde and 4-hydroxybenzaldehyde, respectively.

Anisaldehyde and 4-hydroxybenzaldehyde are two chemicals that can be isolated from plants or via biomass transformation [78,79,80,81]. Conversion of these two aldehydes into terpyridines **22** and **23** (Figure 9) is again effected using Kröhnke’s reaction. Furthermore, it is also possible to prepare compound **23** from **22** through methyl-ether cleavage.

**Figure 10:**
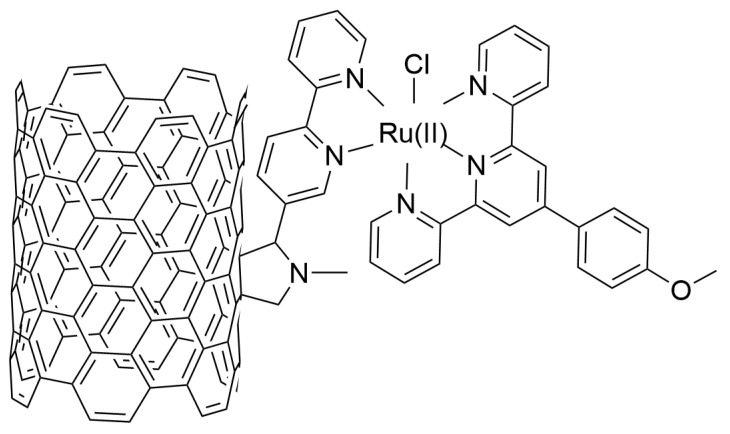
Ru(II)-appended MWCNT-functional material for water splitting to O_2_.

Ligand **22** can be used for the preparation of a Ru(II)-heteroleptic complex that is grafted onto multi-walled carbon nanotubes (MWCNT), thus providing a material (Figure 10) that is able to catalyze water splitting for the production of oxygen [82].

Fabrication of this material is a two-step process starting from 4-methyl-2,2′-bipyridine-4′-carbaldehyde [83]. The latter is first grafted onto the nanotube via a Prato reaction [84]. Then, the metallic center is introduced onto the material through a reaction with Ru(**21**)Cl_3_. Electrochemical measurements indicate that the chloride ligand is replaced by water during the oxygen evolution reaction (OER), thus making the reactant (water) close to the active center. Additionally, the nanostructure of the catalyst makes active centers readily accessible. Finally, the covalent grafting ensures a better transfer of electrons. All these properties combined make this material an efficient catalyst for water splitting.

**Scheme 8:**
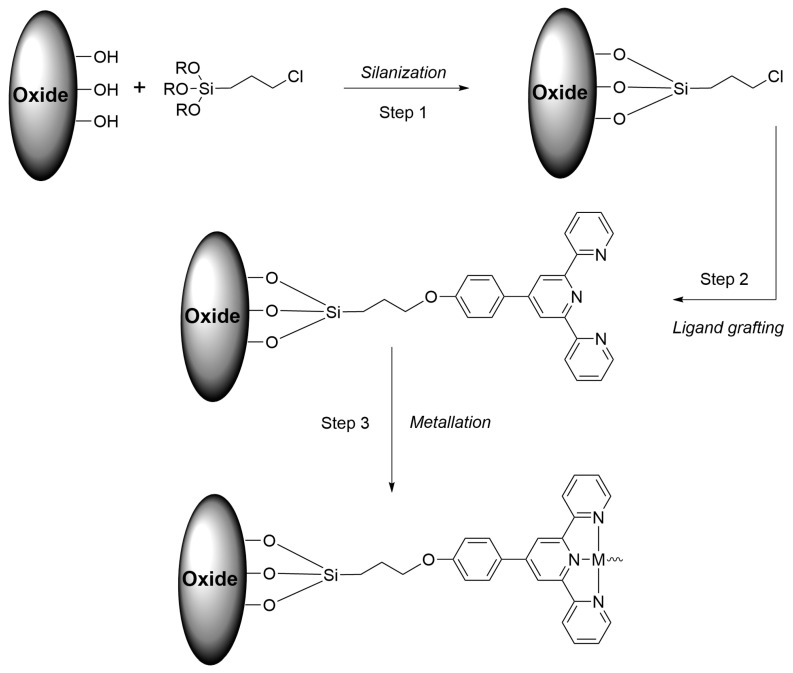
General protocol for the functionalization of various oxides with terpyridine **23**.

Grafting of ligand **23** onto inorganic materials (such as silica or iron oxide) proceeds using the general protocol outlined in Scheme 8. Firstly, the oxide is activated by silanization (step 1) with an alkoxysilane derivative [85]. Especially, (3-chloropropyl)trimethoxysilane is used since it contains a C-Cl bond prone to nucleophilic substitution. The reaction of activated oxide with ligand **23** forms a covalent bond between oxide particles and the terpyridine unit (step 2). Finally, the complexation of the terpyridine unit with various metals (step 3) leads to functional materials for catalytic applications.

This methodology is employed for the preparation of numerous heterogeneous catalysts to be used in many different reactions (Table 1).

**Figure 11:**
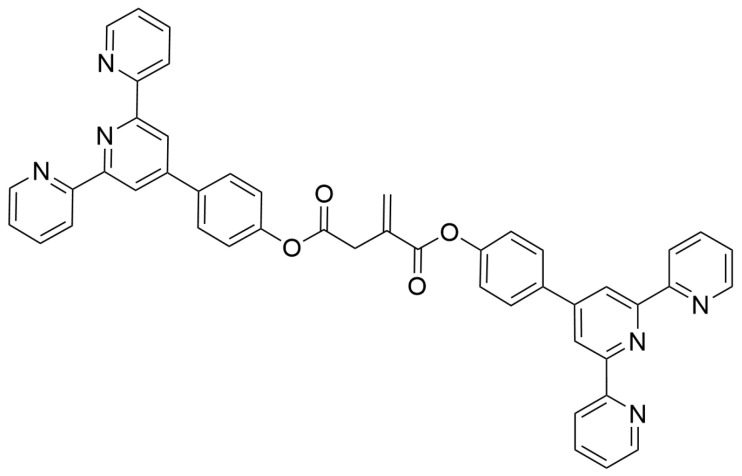
Chemical structure of bis-terdentate ligand **24**.

A similar methodology was employed for preparing a polymer-stabilized palladium catalyst suitable for Suzuki and Heck reactions [91]. This catalyst is loaded onto iron oxide for magnetic recovery. The activation of iron oxide particles is effected with 3-(trimethoxysilyl) propyl methacrylate, leaving a material with pendant reactive acrylate moieties. The latter is reacted with terpyridine **24** (Figure 11), which features a C=C bond, in a radical polymerization reaction. This creates a polymeric network containing pendant terpyridines. This terpyridine-functionalized material is finally complexed with palladium (PdCl_2_). Since the complexation step is carried out in ethanol, Pd(II) is reduced to Pd(0) to afford the active catalyst as Pd nanoparticles. Interestingly, terpyridine **24** is obtained by esterification between biomass-derived ligand **23** and another biomass-derived chemical, namely Itaconic acid [92]. The active material can be recycled up to four times and its efficiency is similar to other Pd-based nanocatalysts.

It is worth mentioning that compound **24** can also be used as a co-monomer with trimethylolpropane triacrylate to prepare a terpyridine-functionalized copolymer without any supporting oxide. This polymeric material can be loaded with palladium to afford a catalyst suitable for C-C coupling reactions [93].

**Scheme 9:**
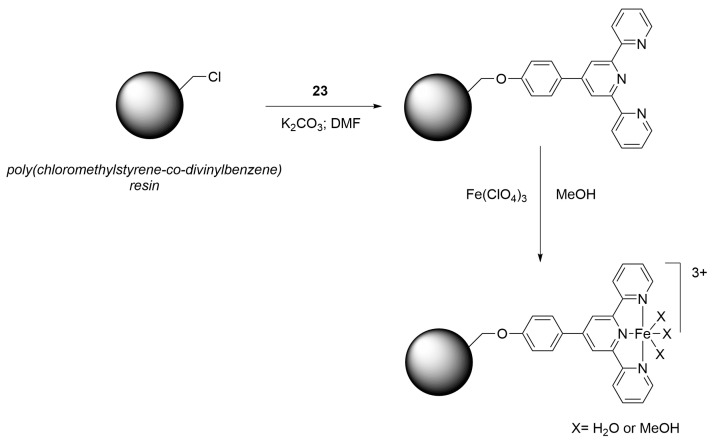
Preparation of a terpyridine complex polystyrene material (perchlorate counter-anions omitted).

Nucleophilic displacement of a chlorine atom onto a is also exemplified in the preparation of an iron terpyridine-containing resin, the latter being used as a catalyst for epoxide opening [94]. A chloromethylated polystyrene-based resin is treated with **23,** thus providing a material with pendant terpyridine units, which are complexed with Fe(III), to afford the catalyst (Scheme 9).

**Scheme 10:**
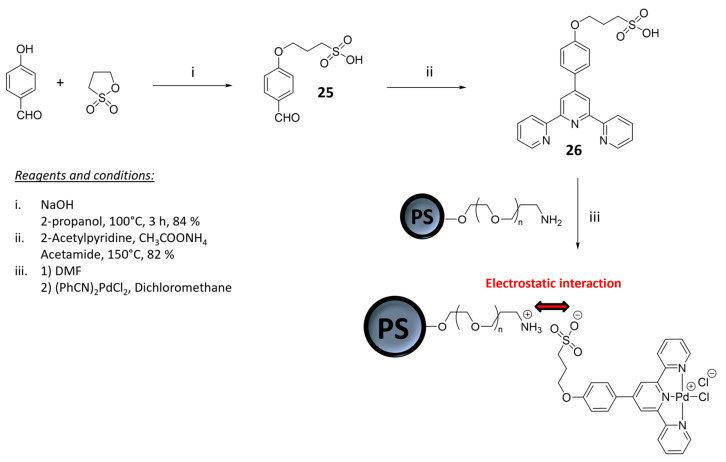
Synthetic pathway towards a PS-terpyridine material suitable as catalyst for C-C coupling.

In the previous examples, the terpyridine system is chemically bonded to the material. Nevertheless, it is also possible to physically bond the ligand through electrostatic interactions. This approach is used to prepare a polystyrene (PS)-based material containing palladium [95]. Firstly, 4-hydroxybenzaldehyde reacts with propanesultone, thus allowing sulfonic acid to contain aldehyde **25**. The latter is then reacted with 2-acetylpyridine to afford ligand **26,** which is reacted with an amino-functionalized PS resin. Proton exchange between the sulfonic acid and the amine group leads to charged species (positively charged PS resin and negatively charged ligand). Electrostatic interactions ensure the anchoring of the ligand onto the material. Finally, complexation with palladium affords an active material suitable for C-C coupling reactions (Scheme 10).

All reactions are carried out in water, making this material an interesting catalyst in view of green chemistry. Furthermore, despite being non-covalently bonded, this material exhibits an interesting chemical stability as demonstrated by the low level of Pd leaching, which remains below 3 μg.L^−1^ (except for the Heck reaction, which shows a small leaching level of 5 ppm).

### 2.3. Metallopolymers, Metal–Organic Frameworks (MOFs) and Polyoxometalates (POMs)

Metallopolymers and MOFs are materials that include coordinated metals in their structures [96,97]. Owing to the ability of terpyridines to easily form complexes with many metals, it is no surprise that such ligands are widely used for the preparation of such materials [98]. Some examples of the utilization of biomass-derived terpy ligands for the construction of MOF or metal-containing polymers have been reported in the literature so far.

**Scheme 11:**
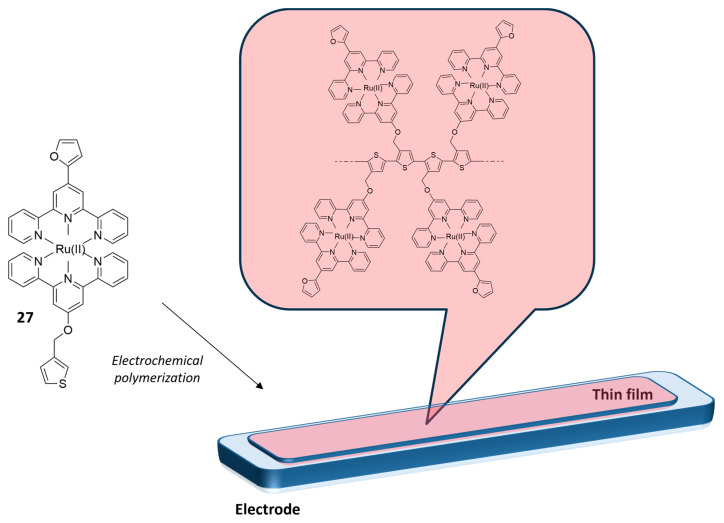
Preparation of a metallopolymer via electropolymerization of complex **27**.

Terpyridine **5** is used for the synthesis of heteroleptic Ru(II) complex **27,** which features a thiophene heterocycle. This complex can be electrochemically polymerized thanks to the presence of the thiophene ring, which provides a material that is deposited onto the surface of an electrode as a thin film (Scheme 11) [99]. The backbone of the material is a polythiophene chain with pendant Ru(II) complexes. Nevertheless, no application for the so-obtained material is reported, as described in this paper, which was solely intended to study the properties of the complex.

**Figure 12:**
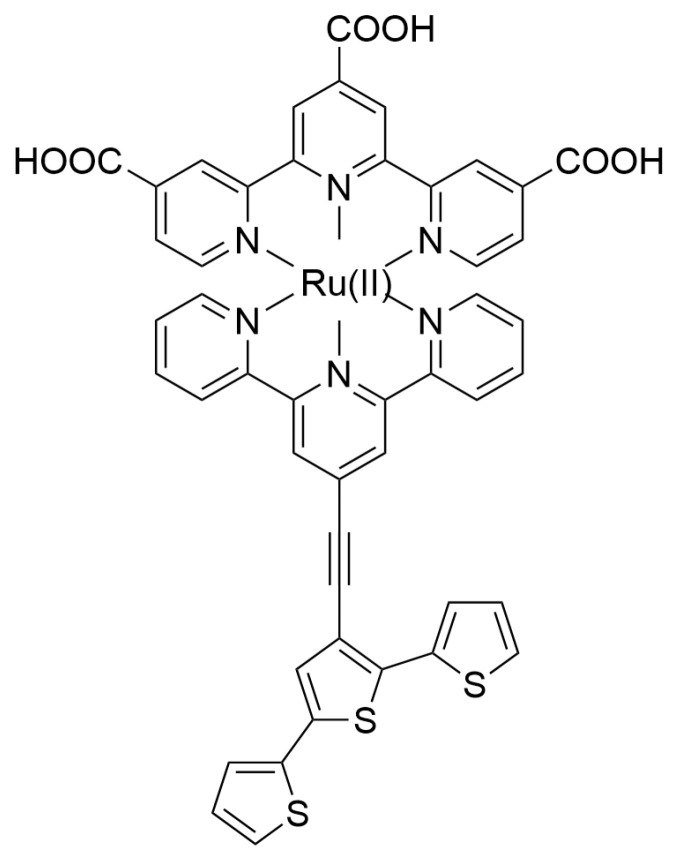
Chemical structure of complex **28**.

Similarly, 4,4′,4″-tricarboxy-2,2′:6′,2″-terpyridine **2** can be combined with a terthiophene-substituted terpyridine to afford a Ru(II) complex (**28**) (Figure 12) that can undergo electropolymerization giving a metallopolymer polymer with pendant Ru(II) metallic centers [100].

The so obtained metallopolymer was tested as a sensitizer in DSSCs but exhibited poor performance. Fast deactivation of the excited state could be the reason for such inefficiency.

**Figure 13:**
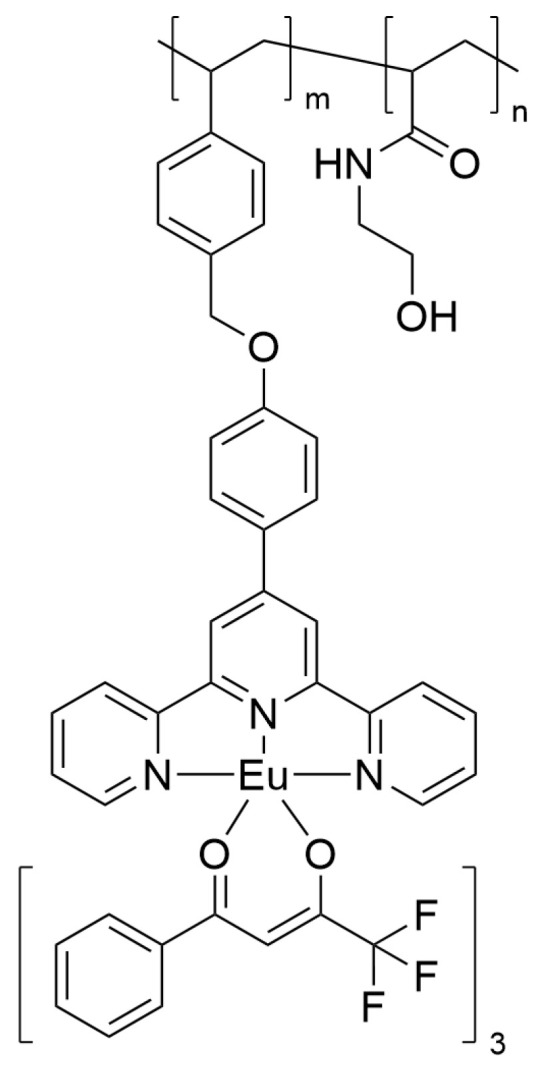
Chemical structure of metallopolymer **29**.

Metal-containing polymer **29** (Figure 13) can be obtained in four steps from 4-hydroxybenzaldehyde. This metallopolymer is water soluble thanks to the presence of -OH groups in the side chains. Owing to the presence of Eu^3+^ ions in its structure, polymer **29** is luminescent. The luminescence decreases in the presence of pyrophosphate anion, thus making this polymer a probe for pyrophosphate monitoring in living cells [101].

The sensing of pyrophosphate anion is affected by measuring the luminescence of the material at λ = 613 nm. The molecular mechanism that is involved is due to the ability of pyrophosphate anions to coordinate with Eu^3+^ ions. This results in the abstraction of Eu^3+^ from the polymer matrix and, in turn, a reduction in the luminescence of the material.

**Figure 14:**
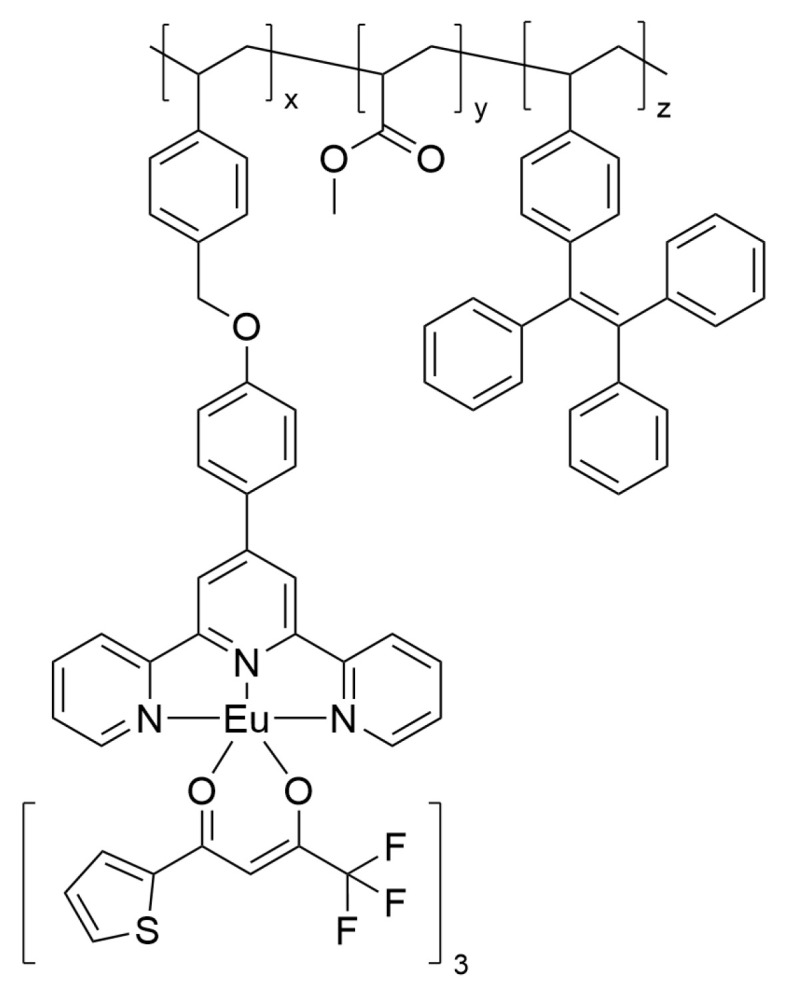
Chemical structure of metallopolymer **30**.

Metal-containing polymer **30** (Figure 14) is obtained in a similar way [102]. It was evaluated as an active material in light-emitting diodes (LED) and as a temperature sensor.

The emissive properties of this material are explained by the transfer of energy between the different constituent parts of the polymer. In particular, transfers between the tetraphenylethene part and the Eu^3+^ ion.

**Figure 15:**
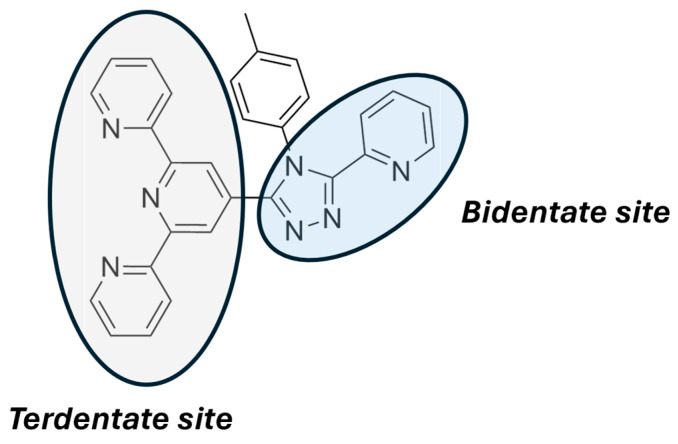
Chemical structure of terpyridine **31**.

The three previous examples are metal-containing polymers where the metal is in a pendant position. However, it is also possible for the metallic centers to be included in the polymer backbone. In these cases, the polymer chain is built up by the formation of coordinative bonds. Synthesis of such a material is generally performed using polytopic ligands, which bear multiple coordination sites [103]. Terpyridine **31** (Figure 15) is a ditopic ligand that is prepared in three steps from 4′-carboxy-2,2′:6′,2′’-terpyridine (**8**).

**Figure 16:**
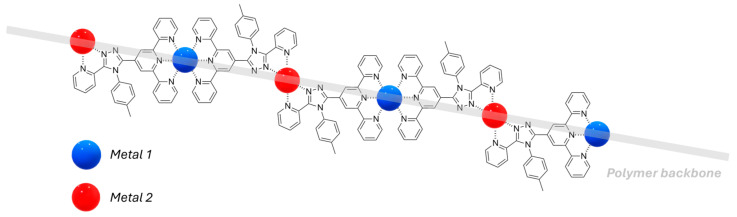
Coordination polymer obtained from ligand **31** and mixed metals.

The originality of compound **31** is the presence of both a terdentate site (terpyridine part of the molecule) and a bidentate site (pyridyl-triazole part of the molecule). This allows the coordination of different metals at each site and the preparation of mixed-metals coordination polymers (Figure 16) [104].

Metal 1 can be selected between Fe(II), Co(II), or Ru(ii), while metal 2 can be either Cu(I) or Ag(I). These metallopolymers were studied for their electrochemical and magnetic properties.

**Figure 17:**
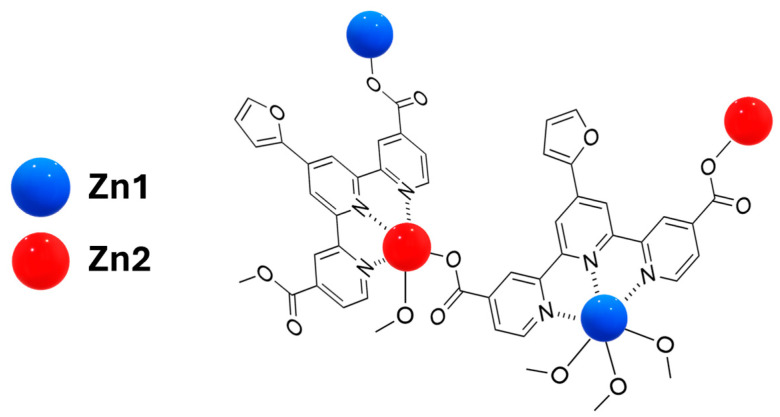
Coordination modes of ligand **4**.

Ligand **4** (Scheme 3) is also considered a polytopic ligand since it possesses the terpyridine scaffold as well as carboxylic acids, which can also be used for coordinating metals. This terpyridine is used for the construction of a 3D porous Zn(II) MOF [105]. The latter is obtained through the complexation of two independent Zn(II) ions in two different coordination modes (Figure 17). The first Zn ion (**Zn1**) is in a distorted octahedral geometry involving the three nitrogen atoms of one molecule of **3** and three oxygen atoms from two other ligands. The second Zn ion (**Zn2**) is five coordinated by using the three nitrogen atoms of one molecule of **3** and two oxygen atoms from two other ligands. The obtained MOF exhibits fluorescence property, which is quenched in the presence of nitro compounds. In fact, the latter can interact with the MOF structure through π–π interactions and/or H-bonding, resulting in fluorescence quenching. Therefore, this MOF could be used as a nitro-compounds sensor.

Additionally, this MOF can be carbonized to afford a porous carbon-based material for gas adsorption [106].

**Figure 18:**
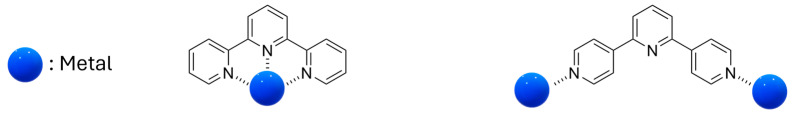
Coordination modes of 2,2′:6′,2″-terpyridines (**left**) and 4,2′:6′,4″-terpyridines (**right**).

All terpyridines described in the present review so far are 2,2′:6′,2″-terpyridine derivatives. Nevertheless, 4,2′:6′,4″-terpyridines, although being less common than 2,2′:6′,2″-terpyridines, are useful bricks for the construction of MOFs or metallopolymers [107,108,109]. Especially 4,2′:6′,4″-terpyridines are used for the coordination of two metallic centers instead of one in the case of 2,2′:6′,2″-terpyridine derivatives. This two metals ligation is effected via the two outer pyridine rings (Figure 18).

**Figure 19:**
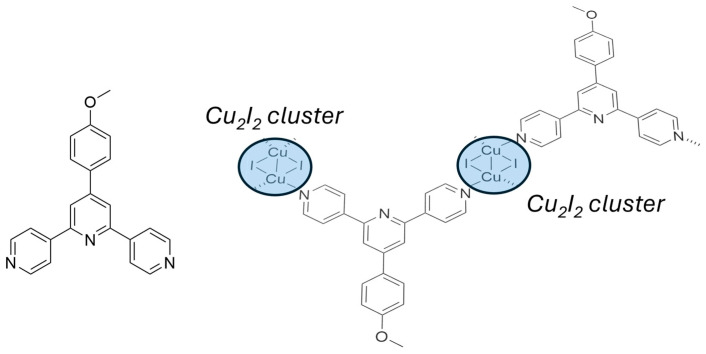
Structure of terpyridine **32** (**left**) and its coordination mode to Cu_2_I_2_ clusters (**right**).

Terpyridine **32** (Figure 19) is obtained from anisaldehyde and 4-acetylpyridine using the classic Kröhnke method [110]. This ligand can be coordinated with CuI, thus forming a 2D MOF with Cu_2_I_2_ clusters as nodes.

The 2D nets so obtained are interdigitated, thus forming a 3D porous MOF with 1D channels. The 3D structure is stabilized by π–π and CH-π interactions, and the voids in the supramolecular structure are able to accept guest molecules. The inclusion of small molecules into the channels leads to the modification of the optical properties of the material, making it an optical sensor for various substances.

Finally, combining terpyridine **32** with various carboxylated co-ligands and Co(II) ions is a strategy that gives rise to various MOF architectures such as 2D sheets, 3D frameworks, and 3D interpenetrated frameworks [111]. These different MOFs were evaluated for their gas adsorption as well as for their magnetic properties.

**Scheme 12:**
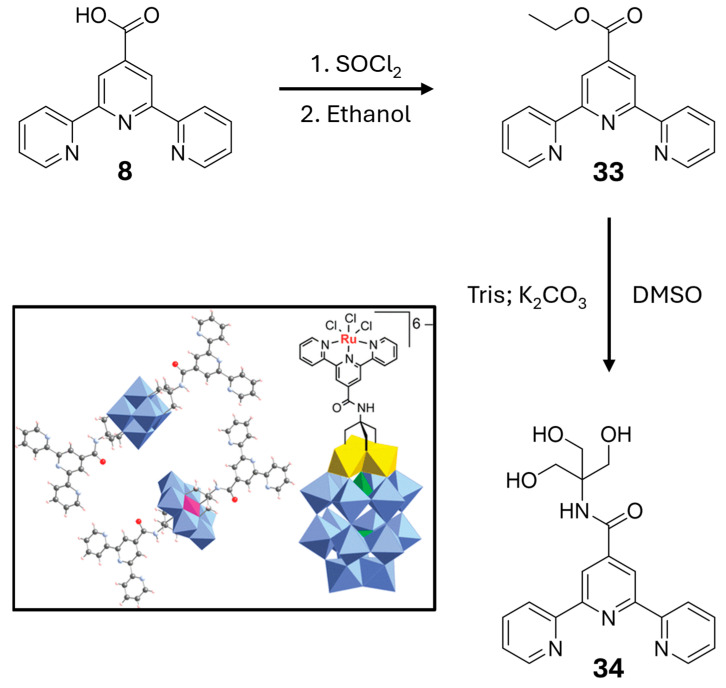
Synthetic pathway towards ligand **34**. Inset: schematic representation of a POM-terpyridine material (adapted from reference [116]).

Polyoxometalates (POM) are inorganic building blocks that possess interesting properties [112]. These materials find applications in many fields, such as catalysis [113], photo(electro-) chemical applications [114], or as sensors [115], just to name a few. POM can be functionalized with a pendant terpyridine moiety using ligand **8** as a starting material [116]. Firstly, compound **8** is converted to ester **33**. Then, the latter is transformed into amide **34** via reaction with Tris. Hydroxy groups onto **34** then allow the grafting of terpyridine moieties onto various POMs (Scheme 12).

The properties of the materials obtained were studied, and although the paper does not describe direct applications of these POMs, a possible one could be as a photoactive material.

## 3. Conclusions and Perspectives

**Figure 20:**
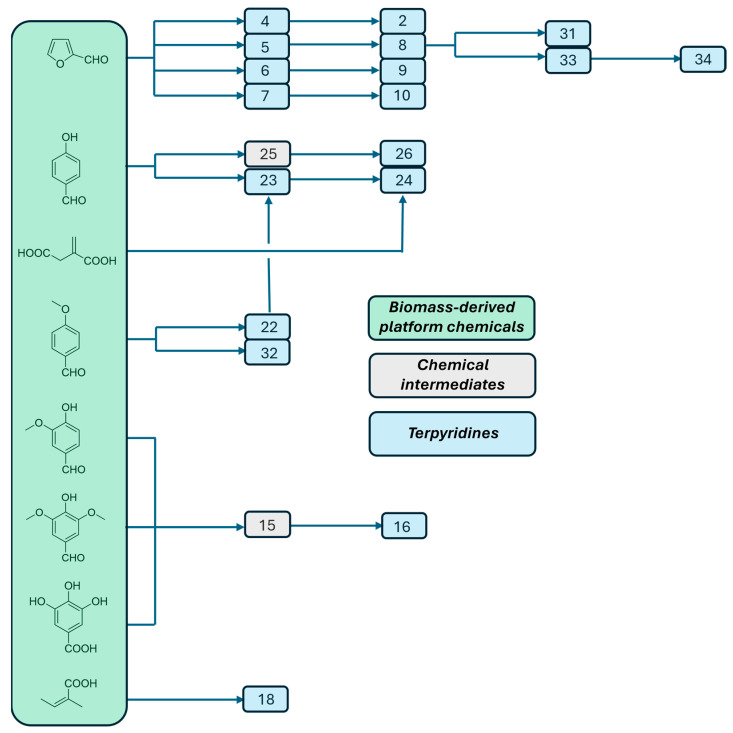
Summary of the different biomass-derived terpyridine ligands discussed in this review and their interconnections.

As demonstrated above, biomass-derived chemicals are versatile reagents for the synthesis of terpyridine ligands to be incorporated into functional materials. Figure 20 highlights the various possibilities that are offered for the preparation of biomass-derived terpyridine ligands.

Given the almost infinite possibilities of compounds that can be obtained by combining a terpyridine ligand with a metal, it is entirely reasonable to believe that biomass-derived terpyridines will continue to be used for the construction of functional materials. Recent scientific literature seems to support this. Indeed, several recently published articles report the use of terpyridines in the manufacture of functional materials, particularly in the field of electrical energy storage [117,118,119,120,121]. Although these recently described ligands are not biomass-derived, it is entirely possible to imagine that the terpyridines shown in the present paper could be incorporated into such functional materials. This is all the more likely as electricity storage issues are widely studied in the context of sustainable development [122] and confirmed by a very recent example of the utilization of terpyridine **2** for the fabrication of a material to be applied in this field [123].

Another perspective for using these biosourced terpyridines could be the valorization of carbon dioxide, again with a view to sustainable development [124]. Indeed, the use of terpyridine-metal complexes in this field of application is already well documented [125,126,127,128,129,130,131,132,133]. Thus, it is reasonable to believe that biosourced terpyridines could play an important role in this area of application.

Finally, the last potential field of applications (i.e., for biomass-derived terpyridines) that will be mentioned in the present paper is the biomedical domain. Many terpyridine-based materials have been evaluated in this field. The latter can be used both for their cytotoxic properties [134,135,136,137], their anti-bacterial properties [138,139], and even as biosensors just to name a few [140,141].

Of course, this list of potential applications is not exhaustive. Many others can be imagined. This is why it is very likely that biomass-derived terpy ligands will continue to be widely studied by the community of chemists and materials scientists in the future.

## Figures and Tables

**Table 1:** Oxide-based heterogeneous catalysts prepared via silanization with (3-chloropropyl)trimethoxysilane and reaction with **23**.

Nature of Oxide	Complexed Metal (M)	Catalyzed Reaction	References
Fe_3_O_4_	Pd	•Suzuki •Heck	[86]
	Cu	•Huisgen •C-S coupling	[87]
MnFe_2_O_4_	Mo	•Epoxidation	[88]
Graphene Oxide	Pd	•Suzuki •Heck •C-N coupling •C-O coupling	[89]
SiO_2_	Nb	•Phtalimides synthesis	[90]

## Data Availability

No new data were created or analyzed in this study.
